# Integrated chromosomal and plasmid sequence analyses reveal diverse modes of carbapenemase gene spread among *Klebsiella pneumoniae*

**DOI:** 10.1073/pnas.2003407117

**Published:** 2020-09-23

**Authors:** Sophia David, Victoria Cohen, Sandra Reuter, Anna E. Sheppard, Tommaso Giani, Julian Parkhill, the European Survey of Carbapenemase-Producing Enterobacteriaceae (EuSCAPE) Working Group, the ESCMID Study Group for Epidemiological Markers (ESGEM), Gian Maria Rossolini, Edward J. Feil, Hajo Grundmann, David M. Aanensen

**Affiliations:** ^a^Centre for Genomic Pathogen Surveillance, Wellcome Genome Campus, Hinxton, CB10 1SA Cambridge, United Kingdom;; ^b^Institute for Infection Prevention and Hospital Epidemiology, Medical Centre, University of Freiburg, 79106 Freiburg, Germany;; ^c^Faculty of Medicine, University of Freiburg, 79106 Freiburg, Germany;; ^d^Modernizing Medical Microbiology Consortium, Nuffield Department of Clinical Medicine, John Radcliffe Hospital, Oxford University, Oxford OX3 9DU, United Kingdom;; ^e^Department of Experimental and Clinical Medicine, University of Florence, 50134 Florence, Italy;; ^f^Clinical Microbiology and Virology Unit, Florence Careggi University Hospital, 50134 Florence, Italy;; ^g^Department of Veterinary Medicine, University of Cambridge, CB3 0ES Cambridge, United Kingdom;; ^h^Milner Centre for Evolution, Department of Biology and Biochemistry, University of Bath, Bath BA2 7AY, United Kingdom;; ^i^Big Data Institute, Li Ka Shing Centre for Health Information and Discovery, Nuffield Department of Medicine, Oxford University, Oxford OX3 7LF, United Kingdom

**Keywords:** *Klebsiella pneumoniae*, carbapenem resistance, carbapenemase genes, plasmids, genomics

## Abstract

Molecular and genomic surveillance systems for bacterial pathogens currently rely on tracking clonally evolving lineages. By contrast, plasmids are usually excluded or analyzed with low-resolution techniques, despite being the primary vectors of antibiotic resistance genes across many key pathogens. Here, we used a combination of long- and short-read sequence data of *Klebsiella pneumoniae* isolates (*n* = 1,717) from a European survey to perform an integrated, continent-wide study of chromosomal and plasmid diversity. This revealed three contrasting modes of dissemination used by carbapenemase genes, which confer resistance to last-line carbapenems. First, *bla*_OXA-48-like_ genes have spread primarily via the single epidemic pOXA-48–like plasmid, which emerged recently in clinical settings and spread rapidly to numerous lineages. Second, *bla*_VIM_ and *bla*_NDM_ genes have spread via transient associations of many diverse plasmids with numerous lineages. Third, *bla*_KPC_ genes have transmitted predominantly by stable association with one successful clonal lineage (ST258/512) yet have been mobilized among diverse plasmids within this lineage. We show that these plasmids, which include pKpQIL-like and IncX3 plasmids, have a long association (and are coevolving) with the lineage, although frequent recombination and rearrangement events between them have led to a complex array of mosaic plasmids carrying *bla*_KPC_. Taken altogether, these results reveal the diverse trajectories of antibiotic resistance genes in clinical settings, summarized as using one plasmid/multiple lineages, multiple plasmids/multiple lineages, and multiple plasmids/one lineage. Our study provides a framework for the much needed incorporation of plasmid data into genomic surveillance systems, an essential step toward a more comprehensive understanding of resistance spread.

## Significance

In many clinically important bacteria, antibiotic resistance genes are primarily carried on plasmids. These can spread horizontally between different strains and species. However, current surveillance systems track chromosomal lineages of bacteria only, leading to an incomplete understanding of how resistance spreads, from within an individual hospital to across country borders. We present an integrated, high-resolution analysis of both chromosome and plasmid sequences using *Klebsiella pneumoniae* isolates sampled during a European survey. We show that carbapenemase genes, which confer resistance to last-line antibiotics, have spread in diverse ways including via one plasmid/multiple lineages (*bla*_OXA-48-like_), multiple plasmids/multiple lineages (*bla*_VIM_, *bla*_NDM_), and multiple plasmids/one lineage (*bla*_KPC_). These different trajectories must be considered in genomic surveillance systems and the design of new interventions.

The incidence of infections due to carbapenem-resistant *Enterobacterales* (CRE) is rapidly rising, posing a major challenge to public health globally (1). Indeed, carbapenem-resistant *Klebsiella pneumoniae*, the most clinically significant member of CRE, was recently highlighted as the fastest-growing resistance threat in Europe in terms of number of infections and attributable deaths (2).

The largest subset of CRE, the carbapenemase-producing *Enterobacterales* (CPE), hydrolyzes carbapenems and other beta-lactam antibiotics using diverse types of beta-lactamase enzymes called carbapenemases (3). Genes encoding these carbapenemases are usually located on plasmids, which can transmit vertically along clonal lineages as well as horizontally between different strains and species (4, 5). Within plasmids, carbapenemase genes are also frequently associated with smaller mobile genetic elements such as transposons and mobile gene cassettes inserted into integrons, extending their recombinatory capability to multiple nested levels (6).

Next-generation sequencing using short-read technologies has vastly improved our ability to unravel the complexities of infectious disease epidemiology. In particular, it has enabled genomic surveillance of high-risk bacterial lineages including tracking of their geographical dissemination (3, 7–9). These surveillance approaches typically use differences in a defined chromosomal region (the “core genome”) that are determined by mapping sequence reads to a reference. However, advances in short-read sequencing have not enabled the same high-resolution tracking of plasmids since typically being mosaic and recombinant, these usually require de novo assembly for accurate comparison. Unfortunately, plasmid assemblies derived from short-read data are usually highly fragmented as a result of numerous repetitive elements (e.g., insertion sequences) and often cannot be distinguished from chromosomal sequences. Recently, these problems have been overcome by the advent of long-read sequencing, which now readily enables complete (or near-complete) and accurate resolution of plasmid sequences, particularly when the data are assembled together with short reads (10, 11). This advance, coupled with the decreasing costs of long-read sequencing, renders large-scale plasmid comparisons increasingly feasible and brings the benefits of the sequencing revolution to bear also on the molecular epidemiology of plasmids.

Despite the rapidly growing databases of carbapenemase-encoding plasmid sequences, no study has systematically analyzed the diversity of these plasmids in clinical isolates across a large, unbiased, and international sample collection. Previously, we analyzed genomes of 1,717 clinical isolates belonging to the *K. pneumoniae* species complex sampled from 244 hospitals in 32 countries during the 6-mo European survey of CPE (European Survey of Carbapenemase-Producing *Enterobacteriaceae* [EuSCAPE]) in 2013 and 2014 (3, 12). Six hundred and seventy-eight (39.5%) carried one or more of the *bla*_OXA-48-like_, *bla*_VIM_, *bla*_NDM_, and *bla*_KPC_ carbapenemase genes. Here, we investigated the diversity of carbapenemase-encoding plasmids among these isolates using combined long- and short-read sequencing of selected representatives. Furthermore, we explored the potential and limitations of using short-read sequence data obtained from all isolates, together with reference plasmids obtained from representatives, to assess the prevalence, distribution, and transmission dynamics of carbapenemase-encoding plasmids across the wider European population. These analyses revealed three major patterns of plasmid transmission that have enabled widespread dissemination of carbapenemase genes.

## Results

### Diversity of the Genetic Contexts of Carbapenemase Genes among *K. pneumoniae*.

Of 1,717 *K. pneumoniae* species complex isolates submitted during the EuSCAPE, we previously found that 249, 56, 79, and 312 carried *bla*_OXA-48-like_, *bla*_VIM_, *bla*_NDM_, and *bla*_KPC_ genes, respectively (3). Eighteen of these carried two genes. In this study, we first analyzed the genetic contexts of these carbapenemase genes in the short-read genome assemblies. The genetic contexts were considered a proxy for plasmid diversity and were used to aid selection of isolates possessing diverse carbapenemase-carrying plasmids for long-read sequencing. In short, assembly contigs containing each of the four carbapenemase genes were clustered into context groups, based on the order and nucleotide similarity of genes flanking the carbapenemase gene (*Materials and Methods* and Datasets S1 and S2). The clustering took account of all genes on the short-read contig sequences, although contigs with fewer than four genes were excluded.

By this criterion, we identified 3, 10, 15, and 45 groups of isolates with different genetic contexts of *bla*_OXA-48-like_, *bla*_VIM_, *bla*_NDM_, and *bla*_KPC_ genes, respectively (Table 1). Overall, 184 of 696 (26.4%) carbapenemase-carrying contigs could be unambiguously assigned to one of these groups. Assignment rates were higher for isolates carrying *bla*_VIM_ and *bla*_NDM_ and lower for those carrying *bla*_KPC_ and *bla*_OXA-48-like_. In particular, only 4 of 249 (1.6%) *bla*_OXA-48-like_–carrying isolates could be assigned to a context group due to the small size of the contigs, which typically carried only *bla*_OXA-48-like_+/− *lysR* genes.

**Table 1.** Number of isolates assigned to different genetic context groups of the carbapenemase genes using short-read sequencing data

Carbapenemase gene	No. of isolates	Median no. of genes per short-read contig (IQR)	No. (%) of isolates with assigned context group	No. (%) of isolates discarded from clustering*	No. (%) of isolates with ambiguous context^†^	No. of context groups
*bla*_OXA-48-like_	249	2 (2–2)	4 (1.6)	221 (88.8)	24 (9.6)	3
*bla*_VIM_	56	6 (2–7)	39 (69.6)	16 (28.6)	1 (1.8)	10
*bla*_NDM_	79	15 (4–22)	59 (74.7)	6 (7.6)	14 (17.7)	15
*bla*_KPC_	312	23 (18–35)	82 (26.3)	0 (0)	230 (73.7)	45

IQR, interquartile range.

* Isolates were discarded from the clustering either because there were fewer than four genes on the carbapenemase-carrying contig or if the carbapenemase gene was found in the assembly without a start or stop codon.

^†^ Isolates were designated an ambiguous context if the carbapenemase-carrying contig matched multiple different (often larger) contigs.

We selected one isolate from each context group for long-read sequencing, with the exception of one *bla*_OXA-48-like_ group and two *bla*_KPC_ groups for which representative isolates were unavailable. Furthermore, since the above-described process resulted in selection of only two *bla*_OXA-48-like_–carrying isolates, we also selected an additional eight. These had *bla*_OXA-48-like_–carrying contigs with greater than or equal to four genes that, despite not clustering unambiguously into a single context group, matched different combinations of other *bla*_OXA-48-like_–carrying contigs (Dataset S2). They were therefore deemed the most likely to represent different plasmids among the remaining isolates. We also long-read sequenced another four *bla*_OXA-48-like_–carrying isolates, which were positive for two carbapenemase genes and had been selected as representatives of *bla*_KPC_, *bla*_VIM_, or *bla*_NDM_ context groups. Furthermore, one isolate selected as a representative of a *bla*_KPC_ context group also harbored *bla*_VIM_. Finally, we long-read sequenced two *bla*_KPC_-carrying isolates from the same context group to investigate possible within-hospital plasmid transfer since they were submitted from the same hospital but belonged to different sequence types (STs).

### Long-Read Sequencing of Representative Isolates Revealed That Most Carbapenemase Genes Were Plasmid Borne.

We assembled long-read sequencing data together with the previously obtained short reads using Unicycler (10) for 79 isolates, encoding a total of 84 carbapenemase genes (Dataset S3). The total number of contigs in the resulting hybrid assemblies ranged from 2 to 44 (median, 9). In 61 of 79 (77.2%) hybrid assemblies, the largest contig was ≥5 Mb, indicating that all, or most, of the chromosomal sequence assembled into a single contig. The assemblies contained one to eight plasmid replicons (median, four), which are sequences used for defining plasmid incompatibility (Inc) groups (13). Multiple plasmid replicons were commonly found on the same contig, representing fusions between different plasmid types.

We found one copy of each carbapenemase gene in the hybrid assemblies. Five (3× *bla*_OXA-48-like_, 1× *bla*_KPC_, 1× *bla*_VIM_) were located on contigs ranging in size from 3.3 to 5.4 Mb, each representing either a partial or putatively complete chromosomal sequence. The remaining 79 genes were located on contigs ranging in size from 2.5 to 313.6 kb, which are hereafter described as putative plasmid sequences. Indeed, a plasmid origin is supported by the circularization of 44 (55.7%) of these sequences, as well as the identification of plasmid replicons in 65 (82.3%). Of 11 of 79 (13.9%) putative plasmid sequences that neither could be circularized nor contained plasmid replicons, we found additional evidence of a plasmid origin for 10 (*SI Appendix*).

### Dissemination of *bla*_OXA-48-like_ Genes by Rapid Spread of pOXA-48–Like Plasmids across Diverse Lineages.

Among the 14 *bla*_OXA-48-like_–carrying hybrid assemblies obtained, we found the carbapenemase gene in 3 chromosomal sequences (3.3 to 5.4 Mb) and 11 putative plasmid sequences (2.5 to 149.6 kb) (Dataset S3). The two isolates sequenced as context group representatives harbored *bla*_OXA-48-like_ on IncX3 and IncA/C2 plasmids, although we also found IncL/M(pOXA48) (*n* = 3), IncL/M(pMU407) (*n* = 1), and ColKP3 (*n* = 3) plasmids carrying *bla*_OXA-48-like_ among the additional hybrid assemblies. Notably, three IncL/M(pOXA48) plasmids of 61.1 to 63.5 kb showed high structural and nucleotide similarity to a well-described 61.8-kb plasmid, pOXA-48a from strain 11978 (14), which belongs to the pOXA-48–like family (*SI Appendix*, Fig. S1).

We determined the prevalence of the different *bla*_OXA-48-like_–carrying plasmid sequences across all *bla*_OXA-48-like_–carrying isolates in the sample collection (*n* = 249) by mapping the short sequence reads to each of the putative plasmid sequences obtained from the hybrid assemblies (*Materials and Methods*). Importantly, this approach cannot reveal whether there have been insertions or rearrangements relative to the reference plasmid or whether a particular resistance gene (in this case, *bla*_OXA-48-like_) is integrated into the same plasmid or located elsewhere, but nevertheless, it provides an indication of how much of each plasmid backbone is present.

**Fig. 1.**
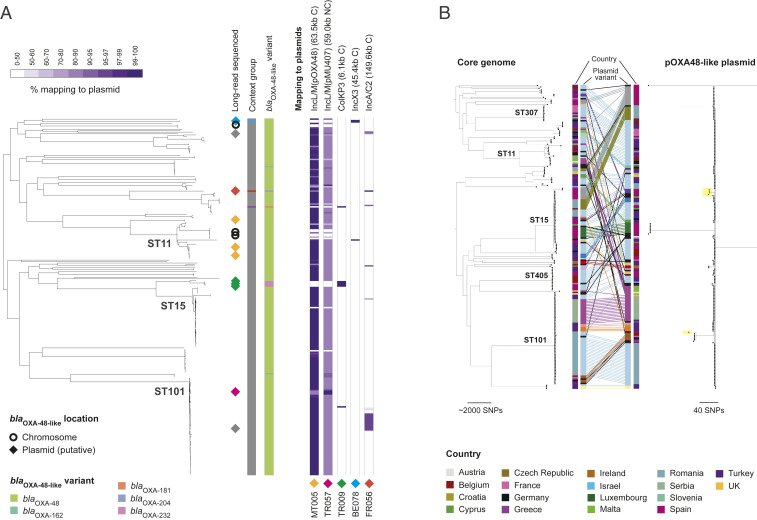
High prevalence of the pOXA-48–like plasmid sequence across *bla*_OXA-48-like_–carrying isolates. (*A*) The phylogenetic tree includes 248 *bla*_OXA-48-like_–carrying isolates from *K. pneumoniae* sensu stricto (the single *bla*_OXA-48-like_–carrying isolate from *K. quasipneumoniae* was excluded). All non–*bla*_OXA-48-like_–carrying isolates, which would be interspersed among the isolates here, were also excluded. Long read-sequenced isolates are marked with a diamond or circle, depending on whether they carry *bla*_OXA-48-like_ on a putative plasmid sequence or the chromosome, respectively. The diamond colors represent distinct plasmids that were obtained. The first two columns, from left to right, show the genetic context group of isolates assigned using the short-read assembly contigs (ambiguous isolates not assigned to any group are in gray) and the *bla*_OXA-48-like_ variant. Remaining columns show the percentage length of *bla*_OXA-48-like_–carrying plasmid sequences obtained from the hybrid assemblies that are mapped by short reads of the 248 *bla*_OXA-48-like_–carrying isolates (note the nonlinear color gradient). Mapping is shown to single representatives of the IncL/M(pOXA48) (i.e., pOXA-48–like) and ColKP3 plasmids since several highly similar plasmids were obtained. Each plasmid sequence is indicated by a diamond of the same color as that indicating the isolate(s) in the tree from which the plasmid was recovered. Mapping data for two shorter *bla*_OXA-48-like_–carrying putative plasmids (20.3 and 2.5 kb) are not shown. C, circular; NC, noncircular. (*B*) The tanglegram links phylogenetic trees constructed using SNPs in the core genome (*Left*) and the pOXA48-like plasmid (*Right*). Both trees are midpoint rooted and include 207 isolates from *K. pneumoniae* sensu stricto that had mapping and bases called (A/T/C/G rather than N) at ≥90% of positions in the plasmid reference sequence. These comprise 202 isolates with *bla*_OXA-48-like_ genes and 5 with *bla*_VIM_ genes, the latter of which are shaded in yellow in the plasmid tree. Lines have been drawn between tips in the trees representing the same isolate, while the tree branches were rotated to minimize the number of overlapping lines required. The lines are colored by the nucleotide sequence variant of the plasmid. Unique plasmid variants are colored black.

Using this approach, we found that the IncX3, IncA/C2, IncL/M(pMU407), and ColKP3 plasmid sequences were found in full only rarely among all 249 isolates (Fig. 1*A* and *SI Appendix*). In contrast, 204 of 249 (81.9%) isolates had short reads that mapped to ≥99% of the circularized 63.5-kb IncL/M(pOXA48) plasmid from EuSCAPE_MT005 (and 221 of 249 [88.8%] to ≥90%). These comprised 202 isolates with the *bla*_OXA-48_ variant and 2 with *bla*_OXA-162_. For non–*bla*_OXA-48-like_–carrying isolates from the sample collection (*n* = 1,468), the median length of mapping to this plasmid was just 2.9% (interquartile range, 2.4 to 4.9%), while only 20 of 1,468 (1.4%) mapped to ≥90% of the plasmid length. Of these 20, we found that 6 actually possessed *bla*_OXA-48-like_ but at a lower coverage than the threshold previously used for determining presence/absence (3). These findings demonstrate a strong association between presence of *bla*_OXA-48-like_ and the pOXA-48–like plasmid. Furthermore, they also explain the poor assignment rates of *bla*_OXA-48-like_–carrying short-read contigs to context groups, stemming from their small size, since the Tn*1999* transposon in which the carbapenemase is contained on this plasmid has consistently precluded contiguous short-read assembly.

Isolates carrying *bla*_OXA-48-like_ and possessing ≥99% of the IncL/M(pOXA48) plasmid sequence belonged to 37 STs across the *K. pneumoniae* species complex and were submitted from 79 hospitals in 19 countries. These findings demonstrate the widespread nature of this plasmid. They also support a high frequency of carriage of *bla*_OXA-48-like_ by the pOXA-48–like plasmid as they rule out the possibility of a spurious association caused by lineage or geographic effects.

Despite the broad distribution of pOXA-48–like plasmids among chromosomal backgrounds, 122 of 204 (59.8%) *bla*_OXA-48-like_–carrying isolates possessing ≥99% of this plasmid sequence belonged to one of three high-risk clonal lineages identified previously (ST11, ST15, ST101, and their derivatives) (3). This is approximately twice the value expected by chance (mean: 29.8%; 95% CI: 29.2 to 30.4%) if the distribution of pOXA-48–like plasmids mirrored the relative abundance of these clonal lineages in the sample collection (*Materials and Methods*).

We next performed phylogenetic analysis of pOXA-48–like plasmid sequences from 202 *bla*_OXA-48-like_–carrying isolates, which included those with both mapped sequence reads and bases called (A/T/C/G rather than N) at ≥90% of reference positions. In the absence of a known outgroup, the resulting phylogenetic tree was midpoint rooted (Fig. 1*B*). The tree highlights the high similarity among these plasmid sequences, with 176 (87.1%) being within two single-nucleotide polymorphisms (SNPs) of each other.

A tanglegram linking the plasmid-based and core genome-based phylogenies shows sharing of plasmid variants between different core genome lineages, providing clear evidence of plasmid horizontal transfer (Fig. 1*B*). This has occurred frequently between core genome lineages that are colocalized at a country level. However, the core genome tree also contains 36 clonal expansions of isolates that each carry a particular plasmid variant, indicative of substantial vertical transmission. The largest of these contains 19 isolates from ST101, submitted from three hospitals across Romania.

Finally, all three hybrid assemblies harboring the *bla*_OXA-48_ variant in the chromosome carried the gene within the Tn*1999* transposon, which itself is contained within a Tn*6237* composite transposon. Tn*6237* is an ∼20-kb sequence that also carries *bla*_OXA-48-like_ in the pOXA-48–like plasmids (*SI Appendix*, Fig. S2). This suggests that mobilization of Tn*6237*, carrying *bla*_OXA-48_, may have occurred from the plasmid into the chromosome. We found evidence of at least two independent chromosomal integrations of Tn*6237* in ST11 and ST530, as well as subsequent clonal spread (*SI Appendix*).

### Spread of *bla*_VIM_ and *bla*_NDM_ Genes Mediated by Transient Associations of Diverse Plasmids with Multiple Lineages.

We obtained hybrid assemblies carrying *bla*_VIM_ genes representing the 10 context groups identified (Dataset S3). Among these, we found *bla*_VIM_ in putative plasmid sequences (46.0 to 284.3 kb) in 9 of 10 hybrid assemblies and in one chromosomal sequence (5.3 Mb). Another putative plasmid sequence harboring *bla*_VIM_ was obtained from an isolate harboring two carbapenemase genes (carrying also *bla*_KPC_) but excluded from further analyses due to the short contig length (2.9 kb). We also obtained hybrid assemblies carrying *bla*_NDM_ genes representing the 15 context groups identified (Dataset S3). All carried *bla*_NDM_ on putative plasmid sequences (12.2 to 197.6 kb). Overall, *bla*_VIM_- and *bla*_NDM_-carrying plasmids harbored diverse replicon types. Several also shared partial structural and/or sequence homology.

**Fig. 2.**
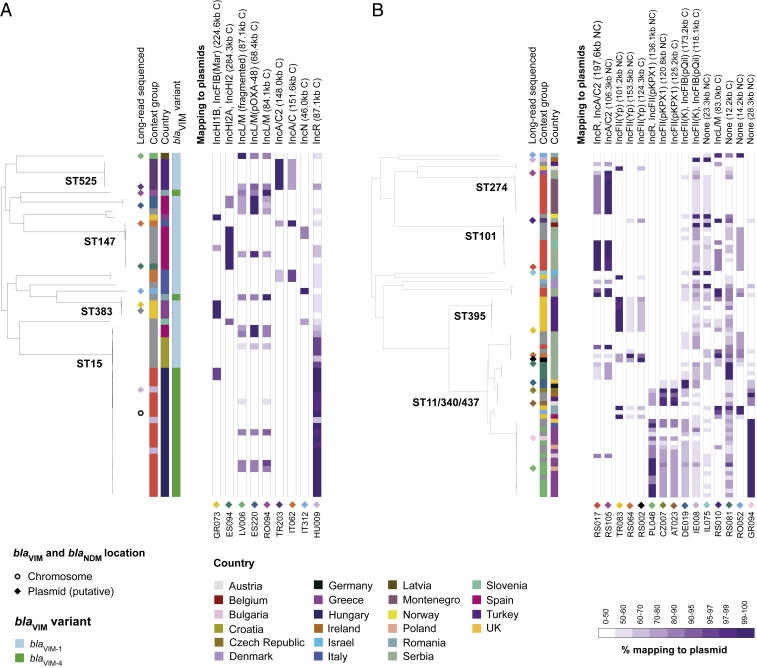
Plasmids carrying *bla*_VIM_ and *bla*_NDM_ genes are associated with individual clonal expansions. The phylogenetic trees show 56 *bla*_VIM_-carrying isolates (*A*) and 79 *bla*_NDM_-carrying isolates (*B*) from *K. pneumoniae* sensu stricto. We excluded all non–*bla*_VIM_ and non–*bla*_NDM_-carrying isolates, respectively, which would be interspersed among the isolates here. Long read-sequenced isolates are marked next to the tree with a diamond or circle, depending on whether they carry the carbapenemase gene on a putative plasmid sequence or chromosome, respectively. The diamond colors represent distinct carbapenemase-carrying plasmids that were obtained. Columns, from left to right, show the genetic context group of isolates assigned using the short-read assembly contigs (ambiguous isolates not assigned to any group are in gray), the country of isolation, and the gene variant (for *bla*_VIM_ genes only as all *bla*_NDM_ genes were *bla*_NDM-1_). Remaining columns show the percentage length of putative plasmids carrying *bla*_VIM_ (*A*) and *bla*_NDM_ (*B*) genes obtained from the hybrid assemblies that were mapped by short reads (note the nonlinear color gradient). Each putative plasmid sequence is indicated by a diamond with the same color as that indicating the isolate in the tree from which the plasmid was recovered. Mapping data for one shorter *bla*_VIM_-carrying putative plasmid are not shown (EuSCAPE_GR075—2.9 kb). C, circular; NC, noncircular.

The same short-read mapping approach used previously allowed us to determine the prevalence of the different plasmid sequences across all *bla*_VIM_- (*n* = 56) and *bla*_NDM_-carrying (*n* = 79) isolates in the sample collection. We found that two *bla*_VIM_-carrying plasmids (from EuSCAPE_LV006 and EuSCAPE_IT312) and one *bla*_NDM_-carrying plasmid (from EuSCAPE_RS064) were each present in only one isolate in the collection since only the individual (long-read sequenced) isolates from which each was derived produced short-read mapping across ≥90% of the plasmid length (Fig. 2). However, many plasmids were associated with clonal expansions of isolates, which were defined as two or more same-ST isolates clustered in the core genome-based phylogeny. In particular, 39 of 56 (69.6%) *bla*_VIM_-carrying isolates belonged to six clonal expansions, and 38 of 79 (48.1%) *bla*_NDM_-carrying isolates belonged to seven clonal expansions, each with ≥99% mapping to a particular plasmid. Isolates from ST11, ST15, and ST101 (and their derivatives) accounted for the majority (56.4 and 71.1%, respectively) of these. Overall, we also found that, in the majority of cases, *bla*_VIM_- and *bla*_NDM_-carrying isolates assigned to a particular context group using short-read data shared the same plasmid sequence obtained from the representative long read-sequenced isolate (although no inferences could be made regarding the structural arrangement).

The core genome diversity within clonal expansions associated with particular plasmids was typically low with maximum pairwise SNP differences per clonal expansion ranging from 0 to 51 (median, 8) for *bla*_VIM_-carrying isolates and from 8 to 54 (median, 17) for *bla*_NDM_-carrying isolates. Using published evolutionary rates for *K. pneumoniae* of 1.9 × 10^−6^ and 3.65 × 10^−6^ SNPs per site per year (15, 16), we estimated that the time taken for two isolates to diverge from a common ancestor by 54 SNPs would be 3.0 to 5.8 y. This is suggestive of recent acquisition of the plasmids by these lineages and indicates that associations between the chromosome and these plasmids may be often only transient. Indeed, four of six and two of seven clonal expansions of *bla*_VIM_-carrying and *bla*_NDM_-carrying isolates, respectively, were restricted to a single hospital (and correspondingly, have few SNP differences). A further two of six and one of seven, respectively, contained isolates submitted from different hospitals in the same country, while four of seven of those with *bla*_NDM_-carrying isolates were from different countries. While isolates from the three high-risk clonal lineages constituted 7 of 13 of the total clonal expansions associated with particular *bla*_VIM_ and *bla*_NDM_ plasmids, they accounted for 6 of 7 of those that had spread to multiple hospitals or countries.

We found some indications of plasmid sharing between STs, with three circularized *bla*_VIM_-carrying plasmids (from EuSCAPE_GR073, EuSCAPE_ES220, and EuSCAPE_RO094) and two circularized *bla*_NDM_-carrying plasmids (from EuSCAPE_IE008 and EuSCAPE_RS010) mapped with short reads across ≥99% of their length by isolates from different STs (Fig. 2). These often included isolates from different STs submitted from the same country but never the same hospital. Notably, they included a 68.4-kb *bla*_VIM-1_–carrying plasmid with high similarity to the pOXA-48–like plasmids that was recovered from the hybrid assembly of an ST483 isolate (EuSCAPE_ES220) but found also in ST11 and ST15 using short-read mapping (Fig. 1*B* and *SI Appendix*, Fig. S3).

While those plasmids shared between lineages were in general found at low prevalence, one exception was a noncircularized 106.3-kb *bla*_NDM_-carrying IncA/C2 plasmid sequence recovered from an ST274 isolate (EuSCAPE_RS105). This was mapped across ≥99% of its length by another eight isolates of the same ST and also by isolates from unrelated STs including ST101 (four isolates), ST147 (two isolates), and ST437 (one isolate). Long-read sequencing of one of the ST101 isolates (EuSCAPE_RS017) demonstrated that this IncA/C2 sequence formed part of a larger 197.6-kb plasmid contig (also noncircularized) comprising both IncA/C2 and IncR replicons. We do not think this hybrid sequence was the result of a misassembly since we found a highly similar contig (191.8 kb) using an alternative long-read assembler, Canu (17), while mapping of the long reads to the 197.6-kb contig also supported its structure. The IncA/C2-IncR plasmid was mapped across ≥99% of its length by six ST101 isolates and an ST147 isolate, some of which also mapped to ≥99% of the shorter IncA/C2 plasmid contig. We could not find the additional sequence from the 197.6-kb plasmid, nor evidence of an IncR replicon, within the assembly of EuSCAPE_RS105. However, only three SNPs were found across 101.4 kb of shared sequence between the two plasmids (*SI Appendix*, Fig. S4), suggestive of recent common ancestry.

### Dissemination of *bla*_KPC_ Genes by Stable Association with ST258/512 Despite Frequent Mobilization between Diverse Plasmids.

We obtained 44 *bla*_KPC_-carrying hybrid assemblies from isolates representing 43 context groups. These included two isolates from the same group selected to investigate possible plasmid transfer between STs (details on these are in *SI Appendix*, Fig. S5). The *bla*_KPC_ gene was found on a chromosomal sequence (3.8 Mb) in one hybrid assembly and on putative plasmid sequences (7.9 to 313.6 kb) in the remaining 43. Twenty-seven of 43 (62.8%) of the putative plasmids, as well as the chromosomal sequence, carried the *bla*_KPC-3_ variant, while the remaining 16 of 43 (37.2%) plasmids carried *bla*_KPC-2_. We found diverse replicon types among the 43 putative plasmids, including those from the single clonal lineage of ST258/512. This lineage contains 230 of 312 (73.7%) of all *bla*_KPC_-carrying isolates in the sample collection.

**Fig. 3.**
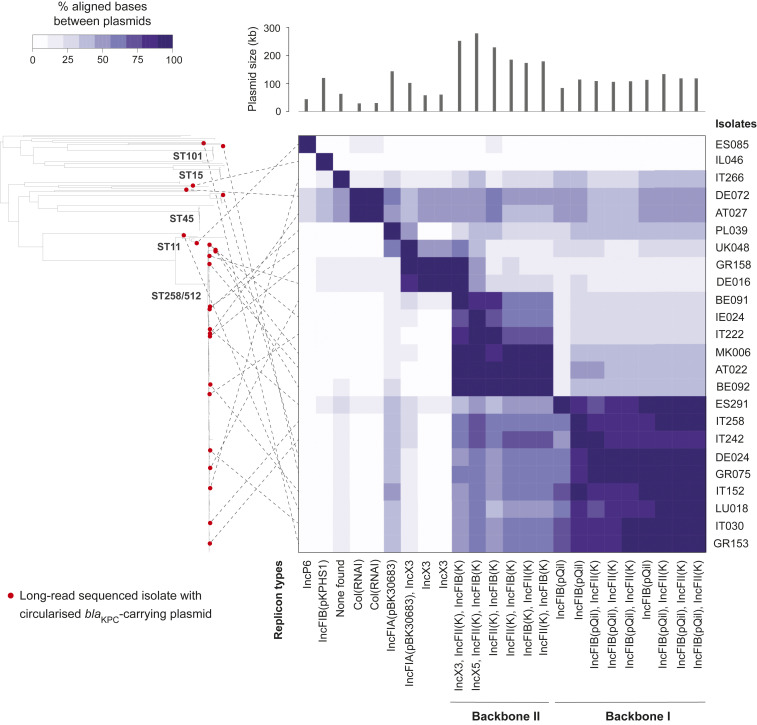
Comparison of 24 circularized *bla*_KPC_-carrying plasmids shows dominance of two major IncF backbone types. The phylogenetic tree contains 311 *bla*_KPC_-carrying isolates from *K. pneumoniae* sensu stricto (the single *bla*_KPC_-carrying isolate from *K. variicola* is excluded). Twenty-four isolates from which circularized *bla*_KPC_-carrying plasmids were obtained are marked by red circles in the tree. The heat map shows the percentage of bases in each plasmid that could be aligned to each of the other plasmids using NUCmer (the row and column orders are the same). Dotted lines link the 24 long read-sequenced isolates in the phylogenetic tree to their respective plasmids in the heat map.

Pairwise sequence comparisons between 24 circularized *bla*_KPC_-carrying plasmids, which we linked to the core genome (chromosomal) phylogeny, demonstrated poor concordance between the plasmid types carrying *bla*_KPC_ genes and their host strains, including within ST258/512 (Fig. 3). It also indicated that 15 of 24 of the plasmids were structural variants of two major IncF backbone types: backbone I (*n* = 9) and backbone II (*n* = 6). The first backbone type represents pKpQIL-like plasmids, and we found that two have an identical size and structure to the originally described pKpQIL plasmid (18) (*SI Appendix*, Fig. S6). The second backbone type shares sequence with pKPN3 (accession no. CP000648) but also pKpQIL-like (backbone I) plasmids (*SI Appendix*, Fig. S7). Both backbone types I and II were found in ST258/512, and each was geographically dispersed. Each of the *bla*_KPC-2_ and *bla*_KPC-3_ gene variants was found on both backbone types. Overall, the finding here that isolates from different context groups harbor *bla*_KPC_-carrying plasmids from the same family highlights the high levels of structural rearrangement, which could lead to rapid further increases in context groupings if applied more widely. The assignment of the two structurally identical pKpQIL-like plasmids to different context groups also provides an example of how inconsistencies in the short-read assemblies may lead to misleading assignments.

Short-read mapping of all *bla*_KPC_-carrying isolates in the sample collection (*n* = 312) to the newly obtained *bla*_KPC_-carrying plasmids demonstrated that some [i.e., the IncP6, IncN, and IncFIB(pKPHS1) plasmids] were found only rarely (*SI Appendix*, Fig. S8). However, many isolates carry two or more of the reference plasmids, including 66 ST258/512 isolates that have ≥99% mapping to four distinct plasmid types with ColRNAI, IncX3, IncFII(K)/IncFIB(pQIL) (i.e., backbone I), and IncFII(K)/IncFIB(K) (i.e., backbone II) replicons. This means that several of the reference plasmid sequences are frequently present in the same isolate, either in the same or a different structural arrangement, and we cannot infer which one (or more) contains the carbapenemase gene using short-read mapping.

We therefore used an alternative approach that takes advantage of *bla*_KPC_ genes typically being on longer contigs in the short-read genome assemblies than other carbapenemases (Table 1). We compared each of the short-read contigs harboring *bla*_KPC_ genes (*n* = 312) with each of the 24 circularized *bla*_KPC_-carrying plasmids from the hybrid assemblies. If ≥98% of the contig sequence could be aligned to a plasmid, we considered this as a match (*SI Appendix*, Fig. S9). We found that 28 of 82 (34.1%) of short-read contigs from non-ST258/512 isolates matched either backbone I or II plasmids. This contrasted with 200 of 230 (87.0%) contigs from ST258/512 isolates. Of these 200, 183 (79.6% of 230) were not compatible with any other plasmid types. These results support backbones I and II (or related variants of these) being the dominant vectors of *bla*_KPC_ genes in ST258/512. However, only 36 of 230 (15.7%) and 28 of 230 (12.2%) ST258/512 contigs could be unambiguously assigned to either backbone I or II, respectively.

**Fig. 4.**
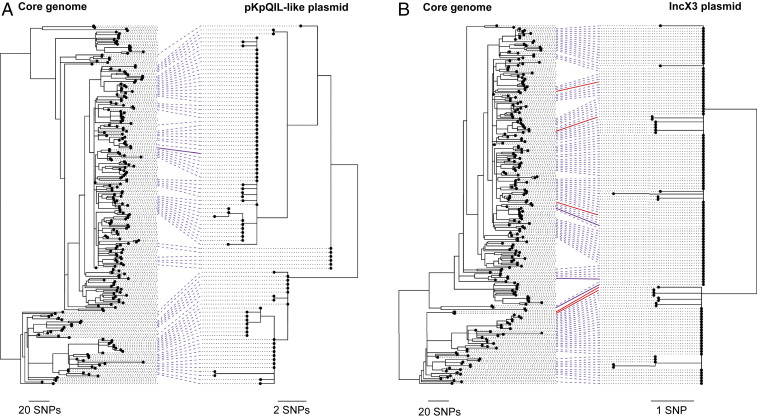
High congruence between pKpQIL-like and IncX3 plasmid phylogenies with the core genome phylogeny of ST258/512 reveals shared evolutionary histories. Each tanglegram comprises a phylogeny of the ST258/512 lineage constructed using all SNPs in the core genome (mapping) alignment and either the pKpQIL-like (*A*) or IncX3 (*B*) plasmids. The core genome phylogenies include all 236 ST258/512 isolates and were rooted using an outgroup. Ninety-one pKpQIL-like and 135 IncX3 plasmid sequences from isolates that had bases (A/T/C/G) called at ≥99% positions in the plasmid reference were included in the plasmid phylogenies. Lines are drawn between tips in the two trees representing the same isolate. The solid purple lines indicate isolates that were found to carry *bla*_KPC_ on a pKpQIL-like (*A*) or IncX3 (*B*) plasmid in the hybrid assemblies. Red lines (in *B* only) indicate isolates that were found to carry *bla*_KPC_ on an alternative plasmid to an IncX3 plasmid in the hybrid assemblies.

We next aimed to understand the evolutionary processes that have led to *bla*_KPC_ genes being carried on diverse plasmids within ST258/512, with a low degree of congruence between the plasmid type carrying *bla*_KPC_ and the core genome-based phylogeny. First, we determined if the plasmids on which we found *bla*_KPC_ are stably associated with the ST258/512 lineage or have been acquired repeatedly from outside of the lineage. We constructed phylogenetic trees of 91 pKpQIL-like plasmids and 135 IncX3 plasmids from ST258/512 isolates. These were from isolates that had short reads that mapped to ≥99% of the plasmid reference sequences and comprised 48.9 and 95.1% of ST258/512 isolates possessing an IncFIB(pQIL) and IncX3 replicon, respectively. Comparisons of these plasmid-based trees with a core genome-based phylogeny of ST258/512 isolates revealed shared evolutionary histories, suggestive of single acquisitions early in the lineage history (Fig. 4). IncX3 plasmid sequences with and without a *bla*_KPC_ gene (as confirmed using the hybrid assemblies) were also intermingled in the phylogenetic tree of IncX3 plasmids, further indicative of vertical propagation of the plasmid within the lineage coupled with occasional gain and/or loss of *bla*_KPC_ (Fig. 4*B*). While phylogenetic reconstructions were not undertaken for the ColRNAI plasmid (due to a lack of diversity) or the backbone II plasmid (due to very high gene content variation), sequence comparisons of these plasmids with and without *bla*_KPC_ genes showed high similarity between their backbones, indicative of a common origin (*SI Appendix*, Figs. S10 and S11). These findings suggest that *bla*_KPC_ genes can be maintained by plasmid backbones that are stable within the lineage. However, they do not reveal whether *bla*_KPC_ genes have moved between plasmids within a single cell or whether they have repeatedly been imported into ST258/512 plasmids from other strains (from either within or outside of ST258/512).

**Fig. 5.**
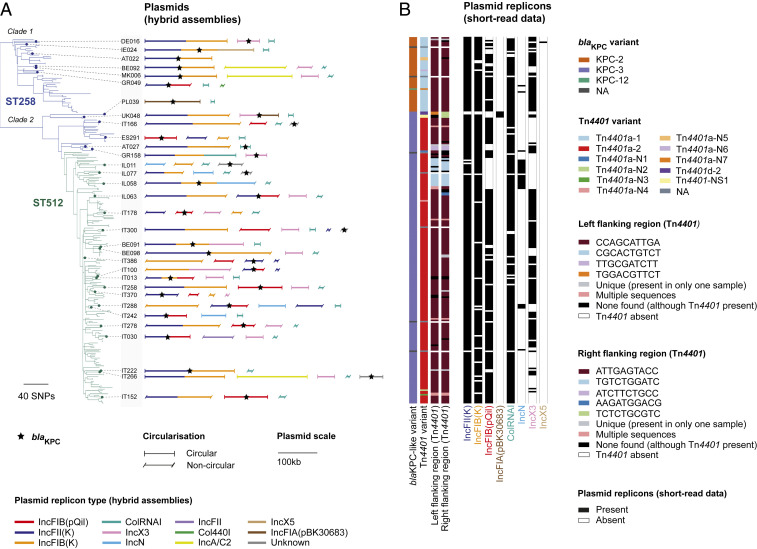
Movement of *bla*_KPC_ genes between plasmids in the ST258/512 lineage. (*A*) The phylogenetic tree contains 236 ST258/512 isolates and was constructed using SNPs from a core genome (mapping) alignment. Thirty-two long read-sequenced isolates carrying *bla*_KPC_ on a putative plasmid sequence are indicated by small circles on the tree tips. Putative plasmid sequences derived from the hybrid genome assemblies with at least one known replicon type and/or containing *bla*_KPC_ are depicted next to the tree. These are scaled by size and colored by any replicon types found in the sequence. A star indicates the presence of *bla*_KPC_ within these sequences. (*B*) Metadata columns, from left to right, show the *bla*_KPC_ variant, the Tn*4401* variant, the 10-bp left and right flanking regions of Tn*4401*, and presence or absence of eight plasmid replicon types that are associated with *bla*_KPC_ in the hybrid assemblies. Tn*4401a*-N1 to Tn*4401a*-N7 represent novel SNP variants of the structural variant, Tn*4401a*. Tn*4401*-NS1 represents a novel structural variant of Tn*4401*. NA, not applicable.

To distinguish between these possibilities, we next used the TETyper tool (19), which takes short-read data as input, to screen all *bla*_KPC_-carrying isolates for the ∼10-kb Tn*4401* transposon and investigate its patterns of inheritance. This transposon is known from previous studies to be the major carrier of *bla*_KPC_ genes in *K. pneumoniae*, especially among European strains (20, 21). Tn*4401* sequences were found in 229 of 230 (99.6%) ST258/512 isolates harboring *bla*_KPC_ genes and classified into “combined” variants based on both structural and SNP variation. We found two predominant combined variants, Tn*4401*a-1 (*n* = 42) and Tn*4401*a-2 (*n* = 176), which differ by a single SNP that also distinguishes the *bla*_KPC-2_ and *bla*_KPC-3_ gene variants. These variants correlate well with the core genome-based phylogeny of ST258/512, with 42 of 46 (91.3%) isolates in one major clade (clade 1) carrying Tn*4401*a-1 and 175 of 184 (95.1%) isolates in the second major clade (clade 2) carrying Tn*4401*a-2 (Fig. 5). This indicates a single major acquisition of Tn*4401* (carrying *bla*_KPC_) by an ancestor of this lineage, followed by relatively stable association during the clonal expansion of ST258/512. Taken together, the combined stability of both the plasmids and Tn*4401* transposon within the ST258/512 lineage suggests that Tn*4401* (carrying *bla*_KPC_) has moved primarily between plasmids in the same bacterial cell or between genetically identical cells (such as those in a clonal infection). We also cannot rule out movement of Tn*4401* between plasmids from different strains, provided that these strains are from the same major clade of ST258/512 (i.e., possess the same combined variant of Tn*4401*). However, the data are not compatible with frequent movement of Tn*4401* between strains from different major clades or frequent import of Tn*4401* into ST258/512 from outside of the lineage.

Finally, we investigated whether Tn*4401* (and *bla*_KPC_) has moved between plasmids via transposition or as part of larger recombination events. Using the short-read data with TETyper, we found identical 10-bp flanking regions (CCA​GCA​TTG​A/ATT​GAG​TAC​C) upstream and downstream of Tn*4401* in 176 of 230 (76.5%) ST258/512 isolates, which include 5-bp ATTGA target site duplications (Fig. 5). Among *bla*_KPC_-carrying plasmids from the hybrid assemblies, these particular flanking regions were restricted to backbone I and II plasmids (and three putative plasmid sequences with no known replicons). Taking these flanking regions to be markers of backbone I and II plasmids, these findings further support the dominant role of the two backbone types as vectors of *bla*_KPC_ genes. They also indicate that *bla*_KPC_ genes are typically mobilized between these plasmid types by larger recombination events (i.e., >10 kb), which transfer Tn*4401* together with additional flanking sequence. Indeed, we found a shared 34-kb sequence region around *bla*_KPC_ in plasmids representing backbones I and II that were recovered from closely related isolates (EuSCAPE_GR049 and EuSCAPE_MK006) (*SI Appendix*, Fig. S12). Only two SNPs were found across this region, suggestive of recent transfer, in contrast to several hundred SNPs found across the remaining homologous sequence. Conversely, we found distinct Tn*4401* flanking regions in the ColRNAI, IncX3, and IncFIA(pKB30683) plasmids carrying *bla*_KPC_ genes in the hybrid assemblies, indicative of transposition of Tn*4401* into these plasmids (Fig. 5). We also identified two different flanking regions both upstream and downstream of Tn*4401* in five ST258/512 isolates, suggesting that *bla*_KPC_ is present in two copies. However, none of these five isolates were long-read sequenced to verify this.

## Discussion

Molecular and genomic surveillance systems for bacterial pathogens currently rely on tracking clonally evolving lineages. By contrast, extrachromosomal plasmids, which can spread horizontally between strains and even species (4, 5), are usually excluded or analyzed with low-resolution techniques (such as Inc typing). This is despite plasmids being the primary carriers of antibiotic resistance genes across many key pathogens. Here, we used combined long- and short-read sequencing of isolates from a European structured survey (EuSCAPE) (3, 12) to investigate the diversity, distribution, and transmission dynamics of resistance plasmids in *K. pneumoniae*. We focused on plasmids carrying carbapenemase genes, which confer resistance to carbapenems, a last-line class of antibiotics. We identified three major patterns by which carbapenemase genes have disseminated via plasmids, summarized as using one plasmid/multiple lineages (*bla*_OXA-48-like_), multiple plasmids/multiple lineages (*bla*_VIM_ and *bla*_NDM_), and multiple plasmids/one lineage (*bla*_KPC_). Despite these contrasts, our work revealed the high dependency of all three modes of carbapenemase gene spread on a small number of high-risk clones.

Previous studies have demonstrated a dominance of high-risk clones among antibiotic-resistant *K. pneumoniae* infections (22), although the reasons driving their success are still debated (23). Here, we have shown that carbapenemase-carrying plasmids are acquired by diverse lineages, such as the pOXA-48–like plasmid that was found in 37 STs. Yet, our phylogenetic analyses indicate that carbapenemase-carrying plasmids are 1) nonrandomly associated with high-risk clones (i.e., ST11, ST15, ST101, ST258/512), 2) propagating by clonal expansion, and 3) frequently spreading across health care networks and national borders. These findings reinforce the importance of preventing transmission, particularly of high-risk STs, through early detection and rigorous infection control.

The first *bla*_OXA-48_–carrying isolate described in 2004 (24) was later found to carry the carbapenemase gene within an IncL/M pOXA-48–like plasmid (14). Since then, numerous studies have reported this plasmid as the dominant vector of *bla*_OXA-48-like_ genes both within and outside of Europe and in both *K. pneumoniae* and other *Enterobacterales* species (25, 26). It has also since been further distinguished as an IncL plasmid, after IncL and IncM plasmids were found to be genetically distinct and compatible (27). Experiments demonstrated that pOXA-48–like plasmids show very efficient conjugation both within and between bacterial species, helping to explain their predominance (28). Here, our comparative analyses demonstrate a single main acquisition of a *bla*_OXA-48-like_ gene by a pOXA-48–like backbone. They suggest that pOXA-48–like plasmids carrying *bla*_OXA-48-like_ genes have emerged recently yet spread rapidly, as we found them in 79 hospitals in 19 countries across Europe. Most notably, we have shown that, despite frequent horizontal transfer of the plasmid, this onward spread has been primarily driven by the clonal expansion of high-risk STs.

By contrast, we found *bla*_VIM_ and *bla*_NDM_ genes on multiple diverse plasmids, which is concordant with reports from the literature (29, 30). As with the pOXA-48–like plasmid, our results show that clonal expansions, especially of high-risk STs, have driven the spread of these plasmids. We noted that associations of clonal lineages with plasmids carrying *bla*_VIM_ and *bla*_NDM_ genes were mostly recent, suggesting that they may be typically only transient. While the maximum age of any clonal expansion associated with a particular plasmid was estimated to be 5.8 y, most were much younger than this. We also found that plasmid sharing between lineages was coupled with structural changes in the plasmids that accumulate over time. We propose that high rates of recombination and rearrangement among plasmids could partially explain both the transient associations between lineages and plasmids, as well as the absence of any single dominant plasmid found across multiple lineages. This capacity of plasmids to undergo rapid structural change also means that plasmid transmission events may go undetected based on crude comparisons of overall plasmid structure and size, and we thus advocate the need for additional comparison of nucleotide diversity over the length of the plasmids.

The most striking example of the reliance on high-risk clones is provided by the *bla*_KPC_ gene. Since its discovery in 1996, this gene has disseminated worldwide at a remarkable pace (22). A single clonal lineage, ST258/512, accounted for >70% of all *bla*_KPC_ genes found among the EuSCAPE sample collection (3). Previous studies have shown that *bla*_KPC_ can be carried by different plasmids in ST258/512 (31, 32). In particular, the pKpQIL-like (backbone I) plasmids have been highlighted as important vectors in North America, Europe, and the Middle East (33–35). However, the origin of the different *bla*_KPC_-carrying plasmids was unknown. Here, we have shown that several of the key plasmid types carrying *bla*_KPC_ genes, including pKpQIL-like plasmids, are stably associated with the ST258/512 lineage. Our data support a single acquisition of *bla*_KPC_ by an early ancestor of the lineage, followed by movement of the gene between different plasmid types in the same bacterial cell. This was coupled with frequent recombination and rearrangement events between different plasmid types, leading to a complex array of mosaic structures carrying *bla*_KPC_ genes in the ST258/512 lineage. The ability of the *bla*_KPC-like_ gene (on the Tn*4401* transposon) to frequently mobilize between diverse plasmids within the ST258/512 lineage may pose substantial risk to further dissemination of the gene both within and outside of the species, especially should an even more promiscuous plasmid be gained by the lineage. This reinforces the need for intensified surveillance and control of the ST258/512 lineage.

We acknowledge several limitations of our study. First, we required carbapenemase-carrying contigs in the short-read assemblies to have greater than or equal to four genes to be used for defining context groups, which then guided selection of isolates for long-read sequencing. This may have reduced the amount of plasmid diversity captured by disregarding isolates carrying carbapenemase genes within particular repetitive structures. Second, we used only one isolate from each context group for long-read sequencing. This meant we were unable to assess the structural diversity and evolution of plasmids within shorter timescales, such as within clonal expansions of *bla*_VIM_- and *bla*_NDM_-carrying isolates. We could also not confirm the stable presence of the carbapenemase gene on particular plasmids within these clonal expansions. Finally, the use of short-read mapping to reference plasmids had varying levels of utility and appropriateness. While it was useful for identification and phylogenetic analyses of structurally stable plasmids (e.g., the pOXA-48–like plasmid), the common occurrence of mosaic plasmids could make these data difficult to interpret.

In summary, we have highlighted three major modes of carbapenemase gene spread, which will continue to evolve as new interactions form between strains, plasmids, and other genetic elements. This demonstrates the urgent need to expand surveillance both within and beyond clinical settings to include not only bacterial strains but also, plasmids, transposons, and other key mobile genetic elements. This will be vital for a comprehensive understanding of antibiotic resistance spread.

## Materials and Methods

### Clustering of Short-Read Assembly Contigs Carrying Carbapenemase Genes into Genetic Context Groups.

Carbapenemase genes (*bla*_OXA-48-like_, *bla*_VIM_, *bla*_KPC_, and *bla*_NDM_) were detected in the previously generated short-read assemblies (3) using BLASTn (36). A minority of carbapenemase genes were not found in the assemblies despite detection using raw sequence reads. Conversely, carbapenemase genes that were detected in the assemblies but found with low coverage using the raw sequence reads (<0.2× the coverage of the multi-locus sequence typing (MLST) gene with the lowest coverage) were excluded. All remaining contigs carrying carbapenemase genes were extracted from the short-read assemblies and annotated using Prokka v1.5 (37). Contigs with four or more genes (including the carbapenemase) were used in the subsequent clustering analysis.

Annotated contigs containing a particular carbapenemase gene were used as input to Roary v3.11.3 (38) to cluster the genes from different contigs into groups based on their nucleotide identity. Roary was run using a 95% blastp identity threshold and the “-s” flag to prevent genes that are presumed to be paralogous being split into different gene groups. Contigs were excluded if the carbapenemase gene lacked a proper start or stop codon, as detected by Roary.

The remaining contigs were assigned to “clustering groups” based on the order and groupings of genes surrounding the carbapenemase (Dataset S1). Those with the same genes (according to the Roary gene groupings) in the same order around the carbapenemase were assigned to the same clustering group, while those with different genes and/or a different order were separated into different clustering groups. Contigs assigned to the same clustering group could be of different lengths and have different numbers of genes, with some contigs extending beyond others. Contigs could also belong to multiple clustering groups (i.e., if they matched multiple, usually larger contigs that are themselves different). Contigs that belonged to a single clustering group were then assigned to a genetic context group, while those that belonged to multiple clustering groups were identified as having an “ambiguous” genetic context (Dataset S2). This clustering method is equivalent to finding all maximal cliques in a graph (39) and the solutions were obtained using a C++ program (https://github.com/darrenstrash/quick-cliques).

### Culture, DNA Extraction, and Long-Read Sequencing.

Seventy-nine isolates were selected for long-read sequencing (Dataset S3). These include one isolate from each of the context groups, with the exception of one *bla*_OXA-48-like_ group and two *bla*_KPC_ groups for which representative isolates were unavailable. An additional eight *bla*_OXA-48-like_–carrying isolates were also included, while two *bla*_KPC_-carrying isolates from the same context group but different STs were selected to investigate possible within-hospital plasmid transfer.

The samples were grown on MacConkey agar plates at 37 °C overnight, and this was repeated until single colonies were visible on the plates. Single colonies of each sample were grown overnight in 7 mL of low-salt lysogeny broth in a shaking incubator. Three milliliters of this culture was spun down at the maximum number of rpm for 3 min, and the supernatant was discarded. The pellet was resuspended in 500 μL of lysis buffer, incubated at 80 °C for 5 min, and then cooled; 275 μL of 3 M sodium acetate was added to the samples, which were vortexed briefly to mix. They were then spun at the maximum rpm for 10 min. The supernatant was removed and added to clean Eppendorf tubes. Five microliters of ribonuclease A was added, and the samples were incubated for 30 min at 37 °C before cooling. Two hundred microliters of protein precipitate solution (Promega) was added, and the samples were incubated on ice for 5 min before being spun at the maximum rpm for 3 min. Finally, ethanol precipitation was performed.

Library preparation for all isolates was performed using the SMRTbell Template Prep Kit 1.0. Long-read sequencing of 39 isolates was performed on the RSII instrument from Pacific Biosciences using C4/P6 chemistry, and 40 isolates were sequenced on the Sequel instrument using v2.1 chemistry and a multiplexed sample preparation. The Sequel data were demultiplexed using Lima in the SMRT link software (https://github.com/PacificBiosciences/barcoding).

### Long-Read Assembly.

The long-read data were assembled together with the previously obtained short reads for each sample (3) using the hybrid assembler, Unicycler v0.4.7 (10). Default settings were used for all samples with the exception of four (EuSCAPE_IL075, EuSCAPE_DE024, EuSCAPE_ES089, EuSCAPE_TR203). For these, we set the flag “–depth_filter,” which represents the fraction of the chromosomal depth below which contigs are filtered out, to 0.1 since the carbapenemase genes were absent from the assemblies when the default setting of 0.25 was used. Assembly statistics were generated using QUAST v.4.6.0 (40). Assemblies were annotated using Prokka v1.5 (37). Annotated assemblies are available in the European Nucleotide Archive (ENA) (*Data Availability*), and accession numbers can be found in Dataset S3.

Canu v1.9 (17) was also used to generate assemblies using long-read data only, and the resulting contigs were compared with those produced by Unicycler to assess support for particular structures.

### Characterization of Hybrid Assemblies.

Contigs containing the carbapenemase genes were identified in the hybrid assemblies using BLASTn (36). Replicon typing of all contigs was performed using Ariba v2.6.1 (41) with the PlasmidFinder database (13). Galileo AMR (42) was used to further identify and analyze the structure of mobile genetic elements within carbapenemase-carrying plasmids.

### Plasmid Comparisons.

NUCmer v3.1 from the MUMmer package (43) was used to determine the length of sequence that could be aligned between pairs of plasmids and the number of SNPs among the aligned regions. The heat map showing the percentage of aligned bases between pairs of complete *bla*_KPC_-carrying plasmids was generated using the “heatmap.2” function from the “gplots” package (v3.0.1.1) in R v3.6.1 (https://www.r-project.org/). The Artemis Comparison Tool v13.0.0 (44) was used to compare and visualize structural variation between two or more sequences.

### Short-Read Mapping to Plasmid Sequences.

Mapping of the previously generated short sequence reads (3) to putative plasmid sequences obtained from the hybrid assemblies was used to determine the length of the reference plasmid sequence present across isolates. Sequence reads were mapped using Burrows Wheeler Aligner (45), and an in-house pipeline (46) was used to identify SNPs using SAMtools (v1.2) mpileup and BCFtools v1.2 (47). In order to call a base (variant or nonvariant), a minimum of eight high-quality mapped reads (and ≥75% of the reads) was required to match the called base with at least three on each strand. The minimum required base quality was 50, and the minimum required mapping quality was 20. The length of the reference plasmid that was mapped by a minimum of one sequence read in each sample was determined from the binary alignment map (BAM) file. This threshold ensured maximum sensitivity of plasmid detection, at the expense of possible false detections resulting from contaminant reads. However, previous quality control filtering steps applied to the short-read data in this collection (3) should ensure that any contamination is limited. Upon testing of this approach, we found that the short reads of each isolate from which we obtained a hybrid assembly mapped to 99.8 to 100% (median, 100%) of the putative carbapenemase-carrying plasmid sequence from the hybrid assembly.

### Core Genome-Based Phylogenetic Analyses.

Phylogenetic trees comprising 248 *bla*_OXA-48-like_–, 56 *bla*_VIM_-, 311 *bla*_KPC_-,and 79 *bla*_NDM_-carrying isolates of *K. pneumoniae* sensu stricto were each constructed using variable positions within an alignment of 2,539 genes. These represent loci that were found to be “core” genes (i.e., present in at least 95% of isolates within each species of the *K. pneumoniae* species complex) in previous analyses (3). Carbapenemase-positive isolates from other species in the species complex (i.e., *Klebsiella quasipneumoniae* and *Klebsiella variicola*) were excluded. Phylogenetic trees were inferred using RAxML v8.2.8 (48) and midpoint rooted. The same core genome alignment was also used to calculate pairwise SNP differences between isolates.

The phylogenetic tree of the ST258/512 lineage was constructed by mapping short reads from the isolates to an ST258 reference genome [NJST258_1 (49)]. Recombined regions were removed from the pseudogenome alignment using Gubbins v1.4.10 (50), and the variable sites in the resulting alignment were used as input to RAxML v8.2.8 (48). The tree was rooted based on previous phylogenetic analyses of the full sample collection that included outgroups of ST258/512 (3).

Phylogenetic trees were visualized and annotated with metadata columns using the Interactive Tree of Life tool (51). Adobe Illustrator (2017.1.0) was used to add additional annotations as well as merge different parts of the figures together.

### Plasmid-Based Phylogenetic Analyses.

To construct a phylogenetic tree of pOXA-48–like plasmids, we first generated a plasmid alignment comprising *bla*_OXA-48-like_–carrying isolates with bases mapped and called at ≥90% of the reference plasmid from EuSCAPE_MT005. This included plasmids from 203 isolates, although one plasmid sequence from the *K. quasipneumoniae* species was subsequently excluded. An additional five *bla*_VIM_-carrying isolates with bases mapped and called at ≥90% of the reference plasmid were also included in the alignment.

Phylogenetic trees of the pKpQIL-like and IncX3 plasmids from the ST258/512 lineage were also constructed using the alignments generated from mapping of the short reads to reference plasmids. The references used were the pKpQIL-like plasmid of EuSCAPE_IT030 and the IncX3 plasmid of EuSCAPE_IL063. Isolates with ≥99% of bases called at the reference positions were included.

Variable positions in these plasmid alignments, excluding any positions containing an N (rather than A/T/C/G) in greater than or equal to one isolate, were used to infer phylogenetic trees using RAxML v8.2.8 (48). Nucleotide variants of plasmids were determined using the same alignments. Tanglegrams linking the core genome and plasmid phylogenies were generated using the “cophylo” function from the “phytools” package (v0.6–60) in R v3.6.1 (https://www.r-project.org/) and further annotated using Adobe Illustrator (2017.1.0).

### Comparison of the Actual and Expected Proportions of pOXA-48–Like Plasmids in High-Risk Lineages.

We compared the actual proportion of pOXA-48–like plasmids carried among three high-risk clonal lineages (ST11, ST15, ST101) with the expected proportion if the distribution of plasmids reflected the relative abundance of these lineages in the population. Isolates belonging to other STs that have evolved from these three STs were included with them, with the exception of ST258/512. We first determined whether each isolate in the full EuSCAPE sample collection carried a pOXA-48–like plasmid, based on whether the short reads mapped to at least ≥99% of the reference plasmid obtained from the hybrid assembly of EuSCAPE_MT005. The actual proportion of isolates carrying a pOXA-48–like plasmid that belonged to one of the three high-risk lineages was calculated. The pOXA-48–like plasmids were then randomly redistributed across all isolates in the sample collection, and the proportion of pOXA-48–like plasmids in the three high-risk clonal lineages was recalculated. This was repeated 100 times, and the mean and 95% CIs were obtained from these values.

### Replicon Typing Using Short-Read Data.

Replicon typing was performed with short-read data using Ariba v2.6.1 (41) with the PlasmidFinder database (13).

### Comparison of *bla*_KPC_-Carrying Short-Read Contigs with Complete Plasmids with NUCmer.

Each short-read contig carrying a *bla*_KPC_ gene was compared with each of the complete *bla*_KPC_-carrying plasmids obtained from the hybrid assemblies using NUCmer v3.1 (43). Contigs that could be aligned over ≥98% of their length to a complete plasmid were deemed to match that plasmid.

### Characterization of Tn*4401* Variation.

The variation within Tn*4401* and its flanking regions was characterized using TETyper v1.1 (19) taking the short reads of all *bla*_KPC_-carrying isolates as input.

## Supplementary Material

Supplementary File

Supplementary File

Supplementary File

Supplementary File

## Data Availability

All raw long-read sequence data and hybrid assemblies for 79 isolates are available from the European Nucleotide Archive (ENA) (accession no. PRJEB33308 [ERP116089]). Previously published short-read data from 1,717 isolates are available from the ENA (accession no. PRJEB10018 [ERP011196]). We have also provided individual accession numbers for the raw short- and long-read sequence data used to generate the hybrid assemblies, the hybrid assemblies themselves, and the short-read assemblies used to determine the context groups in Datasets S2 and S3.
